# Genetic parameters of rumen fill and motility traits in Holstein dairy cows

**DOI:** 10.3168/jdsc.2026-1061

**Published:** 2026-08-06

**Authors:** A. Soufleri, A. Kougioumtzis, V. Tsiamadis, G. Banos, G.E. Valergakis

**Affiliations:** 1 Laboratory of Animal Production and Environmental Protection, School of Veterinary Medicine, Faculty of Health Sciences, Aristotle University of Thessaloniki, GR-54124 Thessaloniki, Greece; 2 Animal and Veterinary Sciences Group, Scotland's Rural College (SRUC), Scotland, UK EH9 3JG

## Abstract

Animal resilience during early lactation is critical for maintaining feed intake, metabolic stability, and overall performance in dairy cows. Rumen fill and rumen motility are practical indicators of rumen function and energy balance; however, their genetic backgrounds have not been previously quantified. The objective of this study was to estimate genetic parameters for rumen fill score, rumen contraction score, and a composite rumen function score in Holstein cows during the first week postpartum. Data were obtained from 938 cows in 9 Greek herds, with rumen traits recorded on d 1, 2, 4, and 8 after calving. Genetic parameters were estimated using repeatability and random regression models. Moreover, an independent dataset of 1,040 cows was used to validate heritability estimates for d 1 postpartum. Rumen fill score exhibited moderate significant heritability (0.16 ± 0.07) and high repeatability, indicating consistent differences among cows across days. In contrast, heritability estimates for rumen contraction score, and composite rumen function score were not significantly different from zero. Results from the independent dataset confirmed the moderate significant heritability of rumen fill score (0.18 ± 0.08). Parity, season of calving, calving ease, and days from calving significantly affected rumen fill and motility traits. These findings demonstrate that rumen fill score is a heritable and repeatable phenotype reflecting inherent differences in rumen function and physiological resilience during early lactation. In addition to herd management, rumen fill score may therefore serve as a useful indicator trait for genetic selection aimed at improving feed intake, metabolic health, and overall robustness of dairy cows.

## Graphical Abstract

**Figure:**
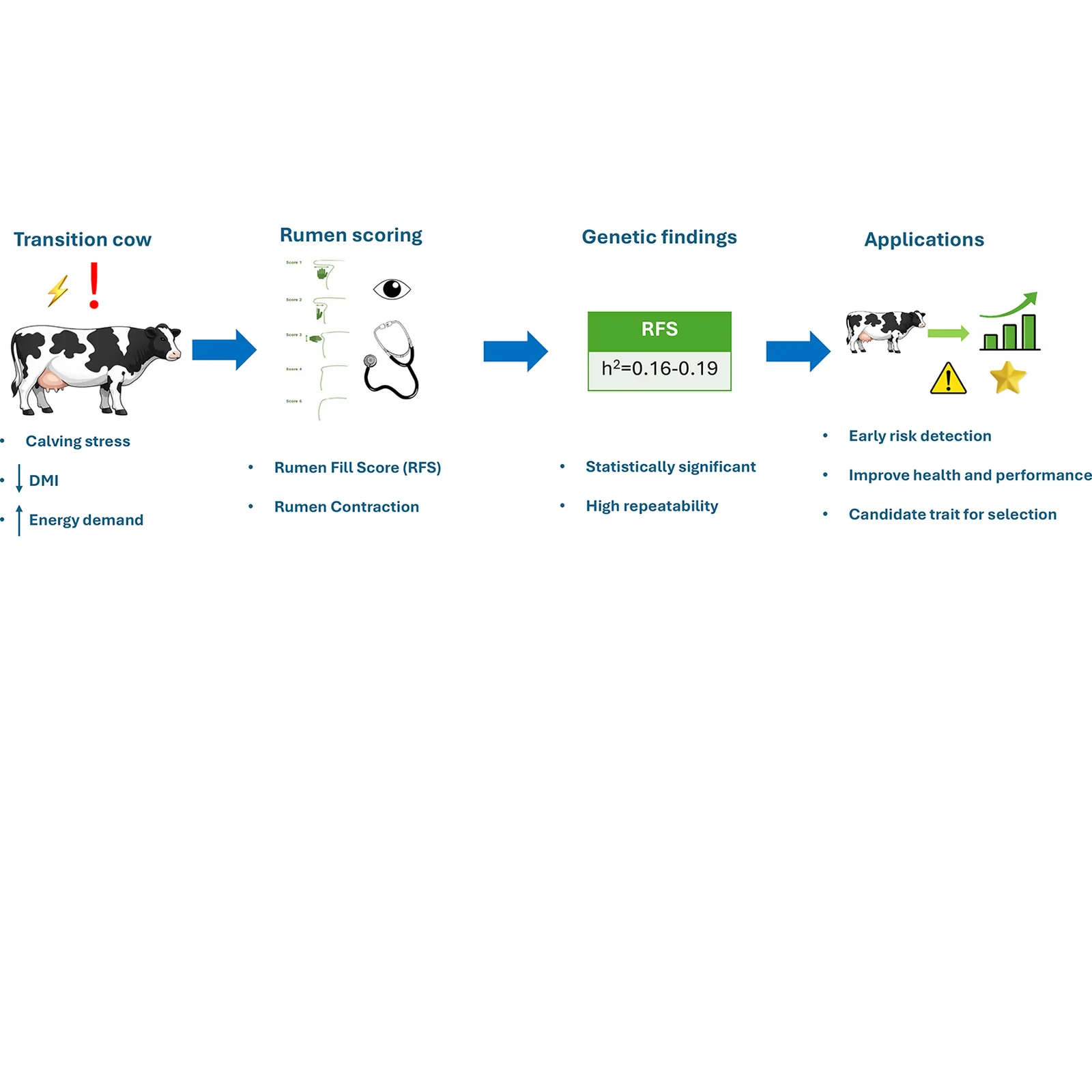

Animal resilience is increasingly recognized as a key determinant of both productivity and sustainability in dairy systems (Friggens et al., 2017). Cows that can sustain feed intake and metabolic stability under stressors such as parturition, abrupt dietary transitions, or environmental fluctuations exhibit superior resilience and reduced susceptibility to metabolic and inflammatory disorders. These animals are better equipped to maintain rumen function, preserve immune responsiveness, and recover more rapidly after parturition, ultimately contributing to improved welfare, longevity, and farm profitability (Friggens et al., 2017).

As dairy cows approach parturition, DMI typically declines by ∼30% (Bertics et al., 1992), coinciding with increased circulating proinflammatory cytokines (Trevisi et al., 2018), which indicate systemic and metabolic stress during the transition period. Moreover, Kvidera et al. (2017) reported that abrupt changes in DMI can compromise gastrointestinal integrity, reducing epithelial barrier function and nutrient absorption. After calving, glucose requirements rise quickly, ∼2.5-fold compared with the dry period (Bell and Bauman, 1997; Drackley, 1999) and increase further if an immune response occurs (Kvidera et al., 2017). Obviously, DMI plays a crucial role to meet these demands. Moreover, cows undergo metabolic and hormonal adaptations, including insulin resistance (de Koster and Opsomer, 2013) and changes in the somatotropic axis (Lucy et al., 2001), which prioritize nutrient allocation for milk production. These adaptations, however, involve extensive mobilization of fat stores, increasing plasma concentrations of nonesterified fatty acids and BHB (Drackley 1999). Excessive lipid mobilization can impair liver function, weaken immune competence, and reduce overall performance, (Sordillo and Aitken, 2009; LeBlanc, 2010; Gross and Bruckmaier, 2019), emphasizing the importance of maintaining adequate DMI to reduce metabolic stress and support health and productivity.

Rumen dynamics play a central role in regulating feed intake and digestion in ruminants. Feed intake is influenced by both physical fill (volume limitation; Allen, 1996) and physiological feedback mechanisms (energy and nutrient signaling; Fisher, 2002; Allen, 2020).

Rumen fill is associated with the observable degree of distension of the left paralumbar fossa, reflecting the amount of digesta present at a given time (Hartnell and Satter, 1979). Because rumen contents are determined by the interplay among intake, digestion kinetics, and passage rate (Llamas-Lamas and Combs, 1991), rumen fill provides a biologically meaningful measure that may capture both short-term feeding patterns and longer-term differences in rumen functionality. Variations in visual rumen fill score within cows are associated with changes in DMI (Burfeind et al., 2010). The trait is generally assessed visually or by palpation of the left paralumbar fossa and scored on an ordinal scale (Hulsen, 2004). Burfeind et al. (2010) confirmed that rumen fill is a reliable measure, showing high intra- and interobserver agreement.

Visual rumen scoring can be applied in dairy herd management as a practical, rapid, and noninvasive tool to monitor nutritional status, appetite, and metabolic disorders in postparturition transition cows (Valergakis et al., 2024). It integrates short-term fluctuations in feed intake and digesta turnover, offering valuable insights into gastrointestinal functionality (Burfeind et al., 2010).

Rumen motility, in contrast, represents the frequency and strength of ruminal contractions that facilitate mixing of ingesta, promote microbial activity, and regulate the passage rate of digesta through the gastrointestinal tract (Grünberg and Constable, 2009). Metabolic disorders such as ruminal acidosis and hypocalcemia, along with diseases that cause pain or fever, can alter the frequency and amplitude of ruminal contractions, leading to impaired motility, digestive disturbances, reduced feed efficiency, and lower production performance (Daniel, 1983; Leek, 1983; Andersen, 2003).

A recent study by Valergakis et al. (2024) provided valuable insights into the biological variability of rumen fill and motility in dairy cows. In their study, postpartum rumen fill and motility scores were associated with subclinical ketosis and subsequent fertility performance. Their findings emphasize that rumen fill is not merely a transient indicator of feeding status but rather a phenotype reflecting inherent differences in gastrointestinal function and energy homeostasis among individuals.

Genetic variation in rumen function has been previously addressed, although the important traits of rumen fill and motility have not been genetically studied. For example, rumination behavior has shown moderate heritability, ranging from 0.30 to 0.45 (Moretti et al., 2016; Lopes et al., 2022; Atashi et al., 2024). Similarly, methane emissions from dairy cattle, a direct result of rumen fermentation, exhibit heritability estimates ranging from 0.12 to 0.45 (de Haas et al., 2011; Lassen and Løvendahl, 2016; Breider et al., 2019). Kamalanathan et al. (2023) reported heritability estimates for methane production, methane yield, and methane intensity of 0.16, 0.27, and 0.21, respectively. van Breukelen et al. (2023) reported heritability estimates for methane-related traits in dairy cows ranging from low to moderate values depending on the measurement method. Furthermore, feed efficiency traits such as DMI and residual feed intake are consistently moderately heritable (Berry and Crowley, 2013; Cavani et al., 2025), suggesting that the physiological determinants underlying rumen fill may similarly possess genetic variation.

From a breeding perspective, identifying cows capable of maintaining rumen fill and high DMI despite the physiological challenges of early lactation could provide valuable insight into the biological basis of resilience. The objective of the present study was to investigate the genetic profile and estimate genetic parameters of rumen fill and rumen motility in dairy cattle.

The main dataset for the present study is described in Tsiamadis et al. (2016), comprising 938 Holstein cows from 9 herds in Greece. The distribution of cows across parities was 434, 232, and 272 for parities 1, 2, and ≥3, respectively. Farms were visited between November 2010 and November 2012 for data collection. Herd management, feeding, and housing were reported in detail (Tsiamadis et al., 2016). Briefly, herd sizes varied from 90 to 300 milking cows, producing on average 8,000 to 12,000 kg of milk per cow per lactation. Animals were milked twice daily and kept in freestall or bedded-pack housing systems. Total mixed rations for dry and fresh cows were balanced to satisfy or exceed NRC (2001) requirements for net energy and metabolizable protein.

All cows were assessed by a veterinarian on d 1, 2, 4, and 8 after calving. Rectal temperature was measured using a digital veterinary thermometer, inserted ∼5 cm into the rectum and left in place until a stable reading was obtained. Simultaneously, BCS was assessed using the 1 to 5 scale of Ferguson et al. (1994) in 0.25-point increments, where 1 indicates emaciated and 5 indicates obese cows. Rumen fill was evaluated after the morning milking while cows were restrained at the feed bunk on each day. Veterinary clinical examination ensured absence of ruminal bloat or pathological rumen distension. None of the cows exhibited clinical signs associated with bloat, including excessive left abdominal distension, increased abdominal wall tension, respiratory compromise, signs of discomfort, or severe impairment of rumen motility. Therefore, the recorded rumen fill scores were considered to represent physiological variation in rumen fill rather than pathological gas accumulation. Rumen fill was scored on a 5-point scale (Hulsen, 2004), based on the depth of the left paralumbar fossa, as described by Valergakis et al. (2024). The rumen fill score (**RFS**) trait was categorized into 3 levels:

RFS1: cows with RFS equal to 1, indicating very low DMI
RFS2: cows with RFS equal to 2, indicating low DMI
RFS3: cows with RFS 3, 4, and 5, reflecting adequate DMI

Rumen motility was assessed immediately following rumen fill scoring by auscultation at the center of the left paralumbar fossa for 2 min using a stethoscope (Divers and Peek, 2017). The number of contractions was recorded. The rumen contraction score (**RCS**) trait was defined as follows:

RCS1: 0–1 contraction per 2 min (impaired rumen motility)
RCS2: ≥2 contractions per 2 min (normal rumen motility)

A composite trait (**RFCS**) combining RFS and RCS was then defined as

RFCS1: all cows with RCS1 (regardless of RFS) and cows with RCS2 and RFS1, indicating unsatisfactory rumen status
RFCS2: cows with RCS2 and either RFS2 or RFS3, indicating satisfactory rumen status (Valergakis et al., 2024)

Blood samples were obtained from each cow on each examination day by coccygeal venipuncture into 10-mL vacuum tubes without anticoagulant (BD Vacutainer, Plymouth, UK) for serum macromineral (calcium, phosphorus, magnesium, and potassium) and BHB measurements. Procedures for sample transport, serum preparation, and BHB analysis are provided in Tsiamadis et al. (2016).

Date of calving, occurrence of twin calving, parity, and calving ease (on a scale 1 to 5, with 1 = quick, easy, and unassisted birth with no intervention needed, and 5 = cesarean section) were recorded for all cows.

A second independent dataset was subsequently considered, comprising 1,040 different cows from 10 herds; herd management, feeding, and housing have also been reported in detail (Soufleri et al., 2019). The distribution across parities was 387, 311, and 342 cows for parities 1, 2, and ≥3, respectively. Farms were visited between February 2015 and September 2016 for data collection. Herd sizes ranged between 90 and 300 lactating cows, with average milk yields of ∼8,000 to 10,000 kg per cow per lactation. Cows were milked twice daily and housed in either freestall or bedded-pack systems. Diets for dry and fresh cows were formulated as TMR to meet or exceed the nutrient requirements for net energy and metabolizable protein as outlined by NRC (2001). Similar data pertaining to d 1 only were available in this second dataset and were used to confirm results obtained from the main dataset analyses.

The main dataset of the present study was first analyzed with a repeatability model to estimate variance components and genetic parameters (heritability and repeatability) for the 3 rumen traits (RFS, RCS, and RFCS). The final model is described as follows:

[1] Y*_ijkq_* = HYS*_i_* + L*_j_* + D*_k_* + A*_q_* + PE*_q_* + e*_ijkq_*,

where Y*_ijkq_* is the dependent variable (rumen trait cow record); HYS*_i_* is the fixed effect of herd-year-season of calving *i* (64 levels); L*_j_* is the fixed effect of number of lactation *j* (4 levels); D*_k_* is the fixed effect of the number of days from calving (*k*, 4 levels); A*_q_* is the random additive genetic effect of individual animal *q*, including all pedigree data (10,263 animals); PE*_q_* is the random permanent environment effect of cow *q*; and e*_ijkq_* is the random residual term.

Subsequently, the same dataset was analyzed with a random regression model to account for covariance between successive records on the same animal. This approach allowed modeling of the longitudinal structure of the data and estimation of variance components and genetic parameters for each day of measurement. This analysis focused exclusively on RFS, as it was the only trait that showed significant heritability in the preceding repeatability model analysis:

[2] $$\text{\textbackslash{}[}\begin{array}{c} Y_{ijmq}=HYS_{i}+L_{j}+\\ \;\sum_{n=0}^{1}b_{m}P_{m}D_{m}+\sum_{n=0}^{1}A_{qm}P_{m}D_{m}+\sum_{n=0}^{1}PE_{qm}P_{m}D_{m}\;+e_{ijmq} \end{array},\text{\textbackslash{}]}$$

where Y*_ijmq_* is the dependent variable; HYS*_i_* and L*_j_* are as in model [1]; P*_m_* is the orthogonal polynomial of order *m*; b*_m_* is the fixed regression coefficient on days from calving (D); A*_qm_* is the random regression coefficient on D associated with the individual animal additive genetic effect including all pedigree data (10,263 animals); PE*_qm_* is the random regression coefficient on D associated with the cow permanent environment effect; e*_ijmq_* is the random residual term; 4 classes were defined here for each day (d 1, 2, 4, and 8) to account for day-to-day variation during early lactation; covariances between the residual classes were assumed to be zero.

In model [2], the final order of the random polynomial was determined using the log-likelihood ratio test through sequential analyses with progressively higher orders. This selection was further supported by the Akaike information criterion.

Finally, the second independent dataset, including only d-1 records, was analyzed with the following model to confirm heritability estimates obtained from the analysis of the main dataset:

[3] Y*_ijq_* = HYS*_i_* + L*_j_* + A*_q_* + e*_ijq_*,

where Y*_ijq_* is the dependent variable (rumen trait record), and all model effects are as in model [1].

The following fixed effect definitions applied to all models. Parity numbers were grouped as 1, 2, 3, and ≥4. Season of calving was categorized winter (December, January, February), spring (March, April, May), summer (June, July, August), or autumn (September, October, November).

Fixed effects were included in the model analyses following preliminary analyses testing their statistical significance (*P* < 0.05, based on *F*-test).

All analyses were performed using the ASReml statistical software package (Gilmour et al., 2015).

Descriptive statistics and heritability and repeatability estimates from the repeatability model [1] for the studied rumen traits are presented in Table 1. Mean values for RFS, RCS, and RFCS were 2.01, 1.81, and 1.65, respectively. The coefficients of variation ranged from 21.74% for RCS to 33.71% for RFS. The heritability estimate for RFS was moderate (0.16 ± 0.07; *P* < 0.05). In contrast, heritability of RCS and RFCS was not different from zero (*P* > 0.05). Repeatability estimates were moderate to high for all traits, ranging from 0.17 ± 0.02 for RCS to 0.54 ± 0.02 for RFS (*P* < 0.05 in all cases).

**Table 1:** Descriptive statistics and trait heritability (h^2^) and repeatability estimates based on the repeatability model analyses (±SE)

Trait^1^	Number of observations	Mean	SD	CV (%)	h^2^	Repeatability
RFS	3,072	2.01	0.68	33.71	0.16 ± 0.07*	0.54 ± 0.02*
RCS	3,134	1.81	0.39	21.74	0.00 ± 0.03	0.17 ± 0.02*
RFCS	3,108	1.65	0.48	28.94	0.00 ± 0.04	0.38 ± 0.02*

1 RFS = rumen fill score; RCS = rumen contraction score; RFCS = combining RFS and RCS.

* *P* < 0.05.

Descriptive statistics for RFS by day postcalving and respective heritability estimates from the random regression model [2] are shown in Table 2. The mean RFS remained relatively stable across the first 4 d postpartum, varying between 1.98 and 2.04. Daily heritability estimates ranged from 0.15 ± 0.07 to 0.19 ± 0.09 (*P* < 0.05) and were consistent with the overall heritability derived from the repeatability model [1]. The estimates in Table 2 account for genetic covariances between successive records on the same individual. Admittedly, however, the short time frame of repeated records (1 wk) suggests that these estimates should be viewed with caution.

**Table 2:** Descriptive statistics and heritability (h^2^) estimates per day after calving for rumen fill score based on the random regression model analysis (±SE)

Days after calving	Number of observations	Mean	SD	CV (%)	h^2^
1	773	2.00	0.72	36.11	0.15 ± 0.07*
2	765	1.98	0.65	33.09	0.19 ± 0.09*
4	764	2.02	0.66	32.91	0.17 ± 0.07*
8	770	2.04	0.66	32.63	0.15 ± 0.09*

* *P* < 0.05.

Parity, season, calving ease, and days after calving had significant effects (*P* < 0.05) on RFS and RCS, illustrated in Figure 1. First-lactation cows exhibited higher RFS compared with cows of higher parity, whereas cows in parity ≥4 showed less favorable RFS and RCS patterns. Both RFS and RCS differed significantly across seasons (*P* < 0.05), with highest values observed in spring and autumn. Cows that experienced easier calvings had higher RFS and RCS than those with more difficult calvings, indicating improved rumen function and emphasizing the effect of calving ease on postparturient physiological status. In addition, both RFS and RCS changed significantly with DIM (*P* < 0.05), reflecting dynamic adaptations in rumen function during the postpartum period.

**Figure 1:**
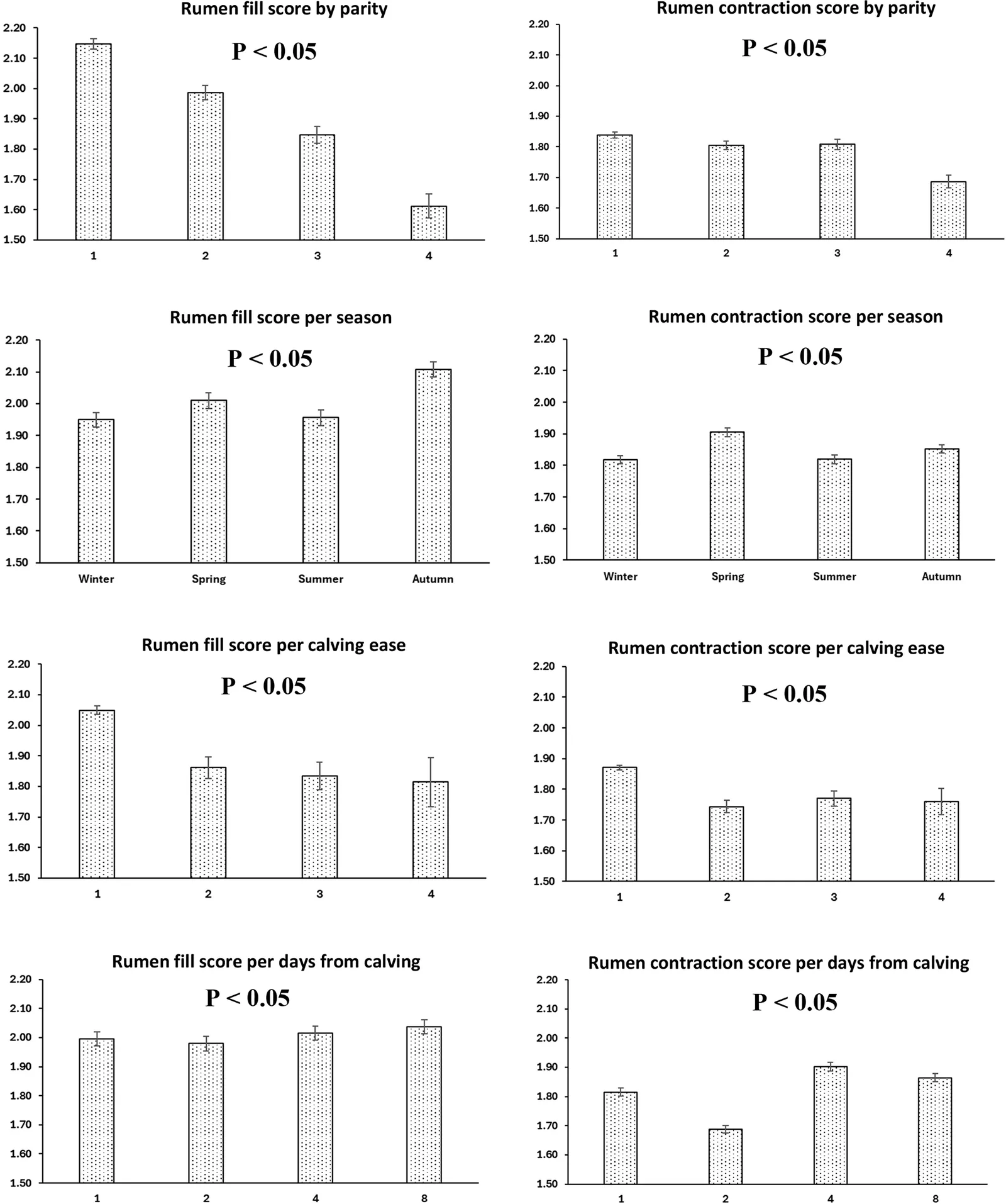
Size of the effects of parity, season, calving ease, and days from calving on rumen fill score (RFS) and rumen contraction score (RCS). The error bars represent SE.

Regarding the independent dataset with only d-1 postcalving data available, the mean values for RFS, RCS, and RFCS were 1.91, 1.80, and 1.54, respectively, with coefficients of variation ranging from 22.51% to 42.28%. The heritability estimate for RFS (0.18 ± 0.08; *P* < 0.05) confirmed the moderate genetic component previously observed. The heritability of RCS and RFCS of 0.00 ± 0.04 and 0.10 ± 0.07, respectively, remained not different from zero (*P* > 0.05).

The present study provides new insights into the inheritance of rumen function traits in dairy cattle during early lactation. To the best of our knowledge, this is the first genetic study of rumen fill and contraction traits in Holstein cattle. The moderate heritability and high repeatability observed for RFS suggest that this phenotype reflects consistent and biologically meaningful differences among cows that extend beyond short-term environmental fluctuations. In contrast, RCS and RFCS showed negligible heritability, indicating that these measures are more strongly influenced by environmental or physiological variation rather than additive genetic effects.

The moderate heritability estimates for RFS (0.16–0.18) obtained in the present study indicate the presence of exploitable genetic variation. It is already known that individual cows differ in traits such as rumen microbiome, feed passage rate, or feeding behavior that are partly under genetic control (Mehtiö et al., 2019; Cavani et al., 2024; Badhan et al., 2025) that may affect DMI. Because rumen fill represents the visible degree of rumen distension, it indirectly captures differences in intake potential and digestive throughput. This aligns with previous work showing moderate heritability for related traits such as feed efficiency, residual feed intake, and methane production (de Haas et al., 2011; Moretti et al., 2016; Cavani et al., 2024). We may therefore speculate that RFS is not merely a visual indicator of current intake, but a complex phenotype linked to the cow's inherent digestive and metabolic efficiency.

By contrast, the low and nonsignificant heritability of the RCS and RFCS traits—inherently more laborious and complex to assess—suggests that rumen motility may be more transient in nature and sensitive to acute factors, including electrolyte balance and metabolic stress. Rumen contractions are known to be inhibited by systemic disturbances such as hypocalcemia, fever, or endotoxemia (Martinez et al., 2014; Vieira-Neto et al., 2024). Consequently, negligible heritability may reflect high within-animal variability and the strong influence of periparturient metabolic challenges, rather than an absence of genetic variation. It is possible that more precise or continuous measurements of rumen motility, such as accelerometer-based rumination sensors (Chang et al., 2022), could reveal higher heritability than the categorical score used here, which resulted from a single observation. The significant repeatability estimates (*P* < 0.05) of the present study offer a promising perspective.

The high repeatability estimates for RFS in early lactation reveal that individual cows maintain consistent differences in rumen fill once they begin to recover from stress around parturition. As the time from calving increases, many cows begin to regain a more stable feeding behavior and rumen environment, while rumination time gradually returns to normal within 7 to 15 d postpartum (Calamari et al., 2014), as inflammation and hormonal perturbations start to subside (Mezzetti et al., 2020). The strong within-cow consistency in RFS suggests that this trait captures a dimension of physiological resilience, implying an ability in certain individuals to maintain feed intake and rumen function despite the metabolic and inflammatory disturbances characteristic of the postcalving period (Friggens et al., 2017).

Cows with higher and more stable RFS in early lactation are likely to experience less negative energy balance, reduced lipid mobilization, and improved liver function. This is consistent with metabolic models of the transition cow, which highlight the importance of maintaining sufficient DMI shortly after parturition as a critical factor in preventing excessive fat mobilization and the onset of ketosis (Wankhade et al., 2017). The moderate but significant heritability of RFS (*P* < 0.05), coupled with high repeatability, highlights the potential of the trait as a genetically and phenotypically meaningful indicator of resilience to transition-period stress.

Low rumen fill and motility early after calving are reportedly unfavorably associated with reproduction and positively associated with hyperketonemia (Valergakis et al., 2024). In the context of these findings, the genetic variation of RFS reported here suggests that selective breeding could improve rumen function, enhance feed intake, reduce the risk of metabolic disorders, and support better future reproductive performance in dairy cows.

The observed heritability of RFS, which was significantly different from zero (*P* < 0.05), also indicates that genetic selection for improved rumen fill could contribute to enhanced robustness and productivity in dairy cattle. Although the visual scoring system used here is simple and cost-effective, its implementation in large-scale breeding programs would require consistent scoring protocols and trained assessors to minimize subjectivity. In the future, rumen fill could be included as part of a broader “resilience index” correlated with feed intake, energy balance, and fertility.

The fact that first-lactation cows had higher RFS than cows of higher parity was somewhat unexpected. These animals are supposed to suffer from social stress (on all farms they were housed together with older cows postpartum), and lower scores were expected. Adult cows had more cases of subclinical hypocalcemia, though (Tsiamadis et al., 2016), and this is a plausible explanation for our observation. The lower RFS and RCS scores observed in winter and summer imply that adverse environmental conditions (cold- and, more importantly, heat-stress) increase the effect of genotype to DMI. As expected, easy calvings were associated with higher scores and, therefore, a less stressful postpartum period. The lowest RFS and RCS observed on d 2 postcalving are probably again linked to the cumulative effect of d-1 and d-2 subclinical hypocalcemia combined with the lowest serum Mg concentrations (Tsiamadis et al., 2016). These results highlight the combined effects of parity, season, calving difficulty, and days after calving on rumen status, underscoring the importance of continuous monitoring of rumen activity after calving.

From a management perspective, RFS may provide a prompt, on-farm diagnostic tool for identifying cows at risk of metabolic disease. Previous studies have shown that low RFS during wk 1 postpartum are associated with subclinical ketosis and reduced fertility (Burfeind et al., 2010; Valergakis et al., 2024). Routine scoring of fresh cows could therefore enable early intervention, such as dietary adjustments or closer health monitoring, to mitigate downstream losses in performance and reproduction.

Despite promising findings, some study limitations should be noted. The categorical nature of rumen fill and contraction scoring may have attenuated the estimated genetic variation. However, relevant continuous variables, although generally better in revealing genetic variation due to higher information content and greater statistical power, are not yet available. In addition, although validation using an independent dataset confirmed the heritability of RFS on the day after calving, future studies should extend analyses beyond wk 1 postpartum to capture adaptation dynamics and long-term stability. The negligible heritability estimates for RCS and RFCS warrant further exploration using alternative phenotypic definitions of rumen motility. Future research should also address trait repeatability estimates across successive lactations, which was not possible in the present study.

Rumen fill is partially under genetic control, reflecting consistent, biologically meaningful differences among cows related to their physiological resilience. In contrast, rumen contraction and motility may be primarily influenced by short-term environmental and metabolic factors. The observed genetic variation in RFS highlights its potential for selective breeding to improve rumen function, feed intake, metabolic health, and reproductive performance. Additionally, RFS provides a practical on-farm indicator metabolic disorder risk, enabling timely interventions during the transition period. These findings support the inclusion of RFS as an animal trait in breeding programs and herd health management practices. Future studies should include genomic analyses and quantification of the relationship of RFS with cow production, health, and fertility. Emphasis should be placed on estimating genetic correlations between RFS and milk production traits, using larger populations and records collected across multiple lactations. Such longitudinal datasets would improve the accuracy of repeatability estimates and provide a more comprehensive understanding of the genetic architecture and biological relevance of RFS throughout the productive life of dairy cows.
